# Potassium 2-iodo­benzene­sulfonate monohydrate

**DOI:** 10.1107/S1600536808019429

**Published:** 2008-07-05

**Authors:** Muhammad Nadeem Arshad, Islam Ullah Khan, Saeed Ahmad, Muhammad Shafiq, Helen Stoeckli-Evans

**Affiliations:** a Materials Chemistry Laboratory, Department of Chemistry, GC University, Lahore 54000, Pakistan; b Department of Chemistry, University of Science and Technology, Bannu, Pakistan; c Institute of Physics, University of Neuchâtel, rue Emile-Argand 11, CH-2009 Neuchâtel, Switzerland

## Abstract

In the crystal structure of the title compound, K^+^·C_6_H_4_IO_3_S^−^·H_2_O, the potasium cation is 2.693 (3)–2.933 (3) Å from the sulfonate and water O atoms (including symmetry-related atoms) and forms a two-dimensional sheet-like structure in the *bc* plane, with the iodo­benzene rings protruding above and below. The water mol­ecule of crystallization is hydrogen-bonded to sulfonate O atoms within this two-dimensional arrangement. Symmetry-related iodo­benzene rings are arranged perpendicular to one another with the I atom *ca* 4.1 Å from the centroid of the neighbouring benzene ring. In the crystal structure, these two-dimensional sheet-like supramolecular structures are arranged parallel to one another, stacked along the *a*-axis direction, with the benzene rings inter­digitated.

## Related literature

For related literature see: Chau & Kice (1977 ▶); Re *et al.* (1999 ▶); Yoshiizumi *et al.*(2004 ▶); Siddiqui *et al.* (2006 ▶, 2007 ▶); Gowda *et al.* (2007 ▶).
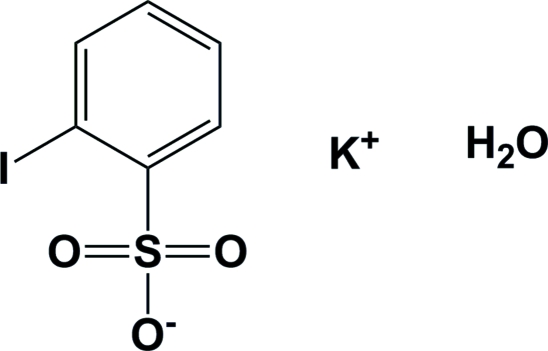


## Experimental

### 

#### Crystal data

K^+^·C_6_H_4_IO_3_S^−^·H_2_O
*M* *_r_* = 340.17
Monoclinic, 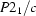

*a* = 13.8993 (4) Å
*b* = 9.0678 (3) Å
*c* = 8.1654 (2) Å
β = 92.260 (2)°
*V* = 1028.33 (5) Å^3^
*Z* = 4
Mo *K*α radiation
μ = 3.70 mm^−1^
*T* = 296 (2) K
0.12 × 0.10 × 0.08 mm

#### Data collection

Bruker Kappa-APEXII CCD area-detector diffractometer
Absorption correction: multi-scan (*SADABS*; Sheldrick, 1996 ▶) *T*_min_ = 0.543, *T*_max_ = 0.754
12144 measured reflections
2804 independent reflections
1961 reflections with *I* > 2σ(*I*)
*R*_int_ = 0.039

#### Refinement

*R*[*F* ^2^ > 2σ(*F* ^2^)] = 0.040
*wR*(*F* ^2^) = 0.113
*S* = 1.00
2804 reflections
118 parameters
3 restraints
H-atom parameters constrained
Δρ_max_ = 1.43 e Å^−3^
Δρ_min_ = −0.68 e Å^−3^

### 

Data collection: *APEX2* (Bruker, 2007 ▶); cell refinement: *SAINT* (Bruker, 2007 ▶); data reduction: *SAINT*; program(s) used to solve structure: *SHELXS97* (Sheldrick, 2008 ▶); program(s) used to refine structure: *SHELXL97* (Sheldrick, 2008 ▶); molecular graphics: *PLATON* (Spek, 2003 ▶); software used to prepare material for publication: *SHELXL97*.

## Supplementary Material

Crystal structure: contains datablocks I, global. DOI: 10.1107/S1600536808019429/cs2082sup1.cif

Structure factors: contains datablocks I. DOI: 10.1107/S1600536808019429/cs2082Isup2.hkl

Additional supplementary materials:  crystallographic information; 3D view; checkCIF report

## Figures and Tables

**Table 1:** Hydrogen-bond geometry (Å, °)

*D*—H⋯*A*	*D*—H	H⋯*A*	*D*⋯*A*	*D*—H⋯*A*
O1*W*—H1*WB*⋯O1^i^	0.86	2.03	2.855 (5)	160
O1*W*—H1*WA*⋯O3^ii^	0.86	2.41	3.266 (5)	179

Symmetry codes: (i) 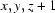; (ii) 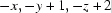.

**Fig. 1.**
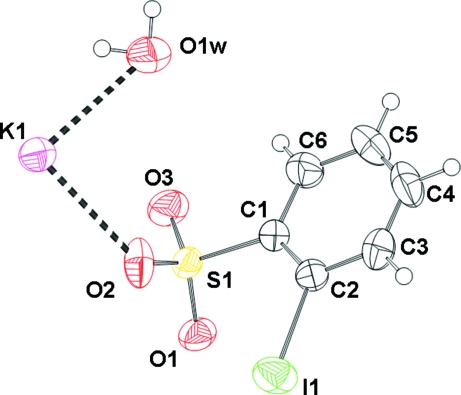
The molecular structure of the title compound, showing the atom numbering scheme and displacement ellipsoids drawn at the 50% probability level [The K···O contacts are shown as a dashed lines].

**Fig. 2.**
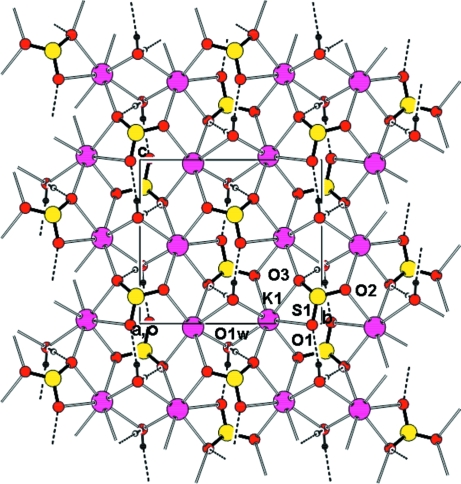
A view down the a axis of the formation of the two-dimensional sheet-like structure formed via the K···O contacts and the O—H···O hydrogen bonds [The hydrogen bonds are shown as dotted lines and the K···O contacts as open bonds. The iodobenzene moieties have been removed for clarity].

**Fig. 3.**
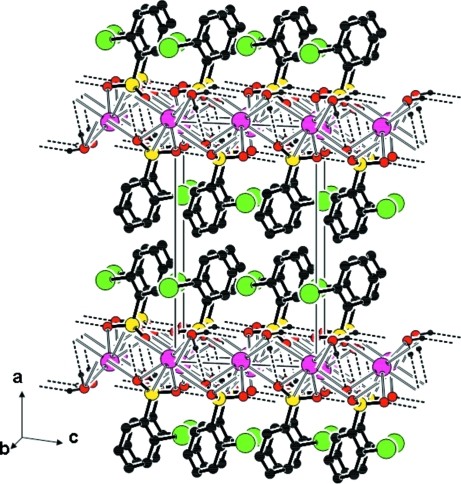
A view along the b axis of the crystal packing of the title compound [The C-bound H-atoms have been removed for clarity].

## supplementary crystallographic information

### Comment

Sulfonic acids belong to an important class of organic compounds particularly the aromatics which have wide applications in different areas (Re *et al.*, 1999). Derivatives of the sodium salt of benzene sulfonic acid were reported as being a scavenger receptor inhibitor (Yoshiizumi *et al.*, 2004). Herein, we report on the crystal structure of the title compound, the potassium salt of 2-iodobenzenesulfoninc acid, in continuation of our research work on the synthesis of biologically active benzothiazine derivatives (Siddiqui *et al.*, 2006, 2007).

The molecular stucture of the title compound is shown in Fig. 1. The bond lengths and angles are similar to those reported for other benzene sulfonates, for example, potassium 4-chlorobenzenesulfonate (Gowda *et al.*, 2007). The potasium cation is between 2.693 (3) to 2.933 (3) Å from the sulfonate and water O atoms (including symmetry related O atoms) and forms a two-dimensional sheet-like structure in the *bc* plane, with the iodobenzene rings protruding above and below (Fig. 2). Symmetry related iodobenzene rings are arranged perpendicular to one another, with the iodine atom *ca* 4.1 Å from the centroid of the neighbouring benzene ring (Fig. 3). The water molecule of crystallization is hydrogen bonded to sulfonate O-atoms (one normal interaction to atom O1, and one rather long interaction to atom O3), within this two-dimensional arrangement (Table 1).

In the crystal structure these 2-D sheet-like supermolecular structures are arranged parallel to one another up the *a* direction, with the benzene rings interdigited (Fig. 3).

### Experimental

The title compound was prepared following the method used by Chau & Kice (1977), and suitable crystals for X-ray analysis were obtained from the reaction mixture.

### Refinement

The water H-atoms were located from a difference Fourier map and initially refined with distance restraints [O—H = 0.88 (2) Å and H···H = 1.45 (2) Å, with *U*_iso_(H) = 1.5*U*_eq_(O)]. In the final rounds of refinement they were held fixed. The C-bond H-atoms were included in calculated positions and treated as riding atoms: C—H = 0.93 Å with *U*_iso_(H) = 1.2*U*_eq_(C). The highest residual density peak, of 1.43 e Å^-2^, is *ca* 0.84 Å from the Iodine atom.

### Crystal data

**Table:**

K^+^·C_6_H_4_IO_3_S^–^·H_2_O	*F*_000_ = 648
*M**_r_* = 340.17	*D*_x_ = 2.197 Mg m^−^^3^
Monoclinic, *P*2_1_/*c*	Mo *K*α radiation λ = 0.71073 Å
Hall symbol: -P 2ybc	Cell parameters from 2742 reflections
*a* = 13.8993 (4) Å	θ = 2.7–24.5º
*b* = 9.0678 (3) Å	µ = 3.70 mm^−^^1^
*c* = 8.1654 (2) Å	*T* = 296 (2) K
β = 92.260 (2)º	Prismatic, green
*V* = 1028.33 (5) Å^3^	0.12 × 0.10 × 0.08 mm
*Z* = 4	

### Data collection

**Table:**

Bruker Kappa APEXII CCD area-detector diffractometer	2804 independent reflections
Radiation source: fine-focus sealed tube	1961 reflections with *I* > 2σ(*I*)
Monochromator: graphite	*R*_int_ = 0.039
*T* = 296(2) K	θ_max_ = 29.3º
φ and ω scans	θ_min_ = 2.7º
Absorption correction: multi-scan(SADABS; Sheldrick, 1996)	*h* = −19→19
*T*_min_ = 0.544, *T*_max_ = 0.754	*k* = −12→12
12144 measured reflections	*l* = −11→11

### Refinement

**Table:**

Refinement on *F*^2^	Secondary atom site location: difference Fourier map
Least-squares matrix: full	Hydrogen site location: inferred from neighbouring sites
*R*[*F*^2^ > 2σ(*F*^2^)] = 0.040	H-atom parameters constrained
*wR*(*F*^2^) = 0.113	*w* = 1/[σ^2^(*F*_o_^2^) + (0.0572*P*)^2^ + 1.0399*P*] where *P* = (*F*_o_^2^ + 2*F*_c_^2^)/3
*S* = 1.00	(Δ/σ)_max_ < 0.001
2804 reflections	Δρ_max_ = 1.43 e Å^−^^3^
118 parameters	Δρ_min_ = −0.68 e Å^−^^3^
3 restraints	Extinction correction: none
Primary atom site location: structure-invariant direct methods	

### Special details

**Table:**

Geometry. Bond distances, angles *etc*. have been calculated using the rounded fractional coordinates. All su's are estimated from the variances of the (full) variance-covariance matrix. The cell e.s.d.'s are taken into account in the estimation of distances, angles and torsion angles
Refinement. Refinement of *F*^2^ against ALL reflections. The weighted *R*-factor *wR* and goodness of fit *S* are based on *F*^2^, conventional *R*-factors *R* are based on *F*, with *F* set to zero for negative *F*^2^. The threshold expression of *F*^2^ > σ(*F*^2^) is used only for calculating *R*-factors(gt) *etc*. and is not relevant to the choice of reflections for refinement. *R*-factors based on *F*^2^ are statistically about twice as large as those based on *F*, and *R*- factors based on ALL data will be even larger.

### Fractional atomic coordinates and isotropic or equivalent isotropic displacement parameters (Å^2^)

**Table:**

	*x*	*y*	*z*	*U*_iso_*/*U*_eq_	
I1	0.31059 (3)	0.31442 (5)	0.46378 (5)	0.0594 (1)	
K1	0.01753 (8)	0.29219 (13)	0.97719 (13)	0.0477 (4)	
S1	0.13443 (7)	0.52155 (13)	0.66539 (13)	0.0349 (3)	
O1	0.1225 (2)	0.5527 (4)	0.4923 (4)	0.0486 (13)	
O1W	0.1268 (3)	0.5113 (5)	1.1461 (4)	0.0617 (16)	
O2	0.1159 (3)	0.3703 (4)	0.7066 (5)	0.0579 (13)	
O3	0.0822 (2)	0.6239 (5)	0.7625 (4)	0.0553 (14)	
C1	0.2582 (3)	0.5494 (5)	0.7203 (5)	0.0340 (12)	
C2	0.3324 (3)	0.4709 (5)	0.6507 (5)	0.0363 (12)	
C3	0.4274 (3)	0.4961 (6)	0.7031 (6)	0.0485 (16)	
C4	0.4485 (4)	0.5972 (7)	0.8240 (7)	0.061 (2)	
C5	0.3762 (5)	0.6753 (7)	0.8924 (8)	0.066 (2)	
C6	0.2817 (4)	0.6523 (6)	0.8416 (7)	0.0516 (17)	
H1WA	0.07210	0.47480	1.17060	0.0930*	
H1WB	0.13980	0.51720	1.24940	0.0930*	
H3	0.47680	0.44410	0.65590	0.0580*	
H4	0.51210	0.61260	0.85960	0.0730*	
H5	0.39090	0.74440	0.97370	0.0780*	
H6	0.23310	0.70620	0.88900	0.0620*	

### Atomic displacement parameters (Å^2^)

**Table:**

	*U*^11^	*U*^22^	*U*^33^	*U*^12^	*U*^13^	*U*^23^
I1	0.0543 (2)	0.0664 (3)	0.0573 (2)	0.0127 (2)	0.0010 (2)	−0.0236 (2)
K1	0.0522 (6)	0.0492 (7)	0.0419 (6)	−0.0032 (5)	0.0056 (5)	−0.0034 (5)
S1	0.0299 (5)	0.0414 (6)	0.0337 (5)	0.0004 (4)	0.0041 (4)	0.0009 (4)
O1	0.0388 (17)	0.074 (3)	0.0328 (16)	0.0053 (16)	0.0004 (13)	0.0044 (15)
O1W	0.071 (3)	0.071 (3)	0.043 (2)	−0.003 (2)	−0.0001 (17)	0.0019 (19)
O2	0.0394 (18)	0.053 (2)	0.081 (3)	−0.0131 (16)	0.0005 (17)	0.018 (2)
O3	0.0424 (19)	0.075 (3)	0.049 (2)	0.0126 (18)	0.0087 (15)	−0.0112 (18)
C1	0.033 (2)	0.035 (2)	0.034 (2)	−0.0023 (17)	0.0020 (16)	−0.0002 (17)
C2	0.037 (2)	0.037 (2)	0.035 (2)	0.0006 (18)	0.0011 (17)	0.0011 (18)
C3	0.035 (2)	0.053 (3)	0.058 (3)	0.001 (2)	0.007 (2)	0.005 (2)
C4	0.041 (3)	0.069 (4)	0.072 (4)	−0.016 (3)	−0.014 (3)	0.000 (3)
C5	0.063 (4)	0.068 (4)	0.065 (4)	−0.021 (3)	−0.010 (3)	−0.020 (3)
C6	0.049 (3)	0.056 (3)	0.050 (3)	−0.003 (2)	0.004 (2)	−0.014 (2)

### Geometric parameters (Å, °)

**Table:**

I1—C2	2.097 (4)	O1W—H1WB	0.8600
K1—O1W	2.827 (4)	O1W—H1WA	0.8600
K1—O2	2.737 (4)	C1—C6	1.390 (7)
K1—S1^i^	3.4125 (16)	C1—C2	1.393 (6)
K1—O1^i^	2.933 (3)	C2—C3	1.391 (6)
K1—O3^i^	2.805 (4)	C3—C4	1.371 (8)
K1—O1W^ii^	2.838 (4)	C4—C5	1.367 (9)
K1—O3^ii^	2.693 (3)	C5—C6	1.378 (9)
K1—O2^iii^	2.711 (4)	C3—H3	0.9300
S1—O1	1.444 (3)	C4—H4	0.9300
S1—O2	1.438 (4)	C5—H5	0.9300
S1—O3	1.436 (4)	C6—H6	0.9300
S1—C1	1.779 (4)		
			
I1···O1	3.407 (3)	O3···H6	2.4200
I1···O2	3.455 (4)	O3···H1WA^ii^	2.4100
I1···K1^iv^	4.1921 (12)	C2···I1^iii^	3.658 (4)
I1···C2^iv^	3.658 (4)	C2···H5^viii^	3.0800
I1···H4^v^	3.3500	C3···H5^viii^	3.0400
K1···I1^iii^	4.1921 (12)	H1WA···O1^vii^	2.7800
O1···I1	3.407 (3)	H1WB···O1^vii^	2.0300
O1···O1W^vi^	2.855 (5)	H4···I1^ix^	3.3500
O1W···O1^vii^	2.855 (5)	H5···C2^x^	3.0800
O2···I1	3.455 (4)	H5···C3^x^	3.0400
O1···H6^viii^	2.8200	H6···O3	2.4200
O1···H1WA^vi^	2.7800	H6···O1^x^	2.8200
O1···H1WB^vi^	2.0300		
			
O1W—K1—O2	86.34 (11)	O3—S1—C1	105.9 (2)
S1^i^—K1—O1W	170.16 (8)	K1^xi^—S1—O3	53.42 (14)
O1^i^—K1—O1W	145.53 (10)	K1^xi^—S1—C1	124.29 (16)
O1W—K1—O3^i^	164.60 (12)	K1^xi^—O1—S1	96.47 (16)
O1W—K1—O1W^ii^	95.19 (13)	K1—O1W—K1^ii^	84.81 (11)
O1W—K1—O3^ii^	72.52 (11)	K1—O2—S1	122.4 (2)
O1W—K1—O2^iii^	78.36 (12)	K1—O2—K1^iv^	99.37 (13)
S1^i^—K1—O2	103.47 (9)	K1^iv^—O2—S1	116.5 (2)
O1^i^—K1—O2	127.91 (11)	K1^xi^—O3—S1	102.30 (17)
O2—K1—O3^i^	80.00 (11)	K1^ii^—O3—S1	154.9 (3)
O1W^ii^—K1—O2	85.40 (12)	K1^xi^—O3—K1^ii^	98.13 (12)
O2—K1—O3^ii^	148.56 (13)	K1^ii^—O1W—H1WB	115.00
O2—K1—O2^iii^	116.37 (13)	K1—O1W—H1WB	128.00
S1^i^—K1—O1^i^	24.87 (7)	H1WA—O1W—H1WB	87.00
S1^i^—K1—O3^i^	24.28 (8)	K1—O1W—H1WA	52.00
S1^i^—K1—O1W^ii^	84.90 (9)	K1^ii^—O1W—H1WA	73.00
S1^i^—K1—O3^ii^	98.14 (9)	S1—C1—C6	118.2 (4)
S1^i^—K1—O2^iii^	97.67 (9)	C2—C1—C6	118.5 (4)
O1^i^—K1—O3^i^	49.09 (10)	S1—C1—C2	123.3 (3)
O1^i^—K1—O1W^ii^	91.88 (11)	I1—C2—C3	116.3 (3)
O1^i^—K1—O3^ii^	77.19 (11)	C1—C2—C3	120.0 (4)
O1^i^—K1—O2^iii^	81.79 (11)	I1—C2—C1	123.7 (3)
O1W^ii^—K1—O3^i^	76.77 (12)	C2—C3—C4	120.3 (4)
O3^i^—K1—O3^ii^	116.67 (10)	C3—C4—C5	120.1 (5)
O2^iii^—K1—O3^i^	114.11 (12)	C4—C5—C6	120.4 (6)
O1W^ii^—K1—O3^ii^	73.97 (11)	C1—C6—C5	120.7 (5)
O1W^ii^—K1—O2^iii^	156.45 (12)	C2—C3—H3	120.00
O2^iii^—K1—O3^ii^	82.50 (12)	C4—C3—H3	120.00
O1—S1—O2	113.6 (2)	C3—C4—H4	120.00
O1—S1—O3	111.9 (2)	C5—C4—H4	120.00
O1—S1—C1	106.95 (18)	C4—C5—H5	120.00
K1^xi^—S1—O1	58.66 (14)	C6—C5—H5	120.00
O2—S1—O3	112.8 (2)	C1—C6—H6	120.00
O2—S1—C1	104.9 (2)	C5—C6—H6	120.00
K1^xi^—S1—O2	130.67 (17)		
			
O2—K1—O1W—K1^ii^	85.04 (11)	O2—S1—O1—K1^xi^	124.5 (2)
O1^i^—K1—O1W—K1^ii^	−100.99 (19)	O3—S1—O1—K1^xi^	−4.7 (2)
O1W^ii^—K1—O1W—K1^ii^	0.00 (10)	C1—S1—O1—K1^xi^	−120.27 (17)
O3^ii^—K1—O1W—K1^ii^	−71.38 (11)	O1—S1—O2—K1	−139.0 (2)
O2^iii^—K1—O1W—K1^ii^	−157.09 (12)	O1—S1—O2—K1^iv^	−16.9 (3)
O1W—K1—O2—S1	−42.7 (3)	O3—S1—O2—K1	−10.3 (3)
O1W—K1—O2—K1^iv^	−172.53 (14)	O3—S1—O2—K1^iv^	111.9 (2)
S1^i^—K1—O2—S1	136.5 (2)	C1—S1—O2—K1	104.5 (2)
S1^i^—K1—O2—K1^iv^	6.62 (12)	C1—S1—O2—K1^iv^	−133.3 (2)
O1^i^—K1—O2—S1	141.7 (2)	K1^xi^—S1—O2—K1	−70.9 (3)
O1^i^—K1—O2—K1^iv^	11.79 (19)	K1^xi^—S1—O2—K1^iv^	51.3 (3)
O3^i^—K1—O2—S1	130.2 (3)	O1—S1—O3—K1^xi^	5.0 (2)
O3^i^—K1—O2—K1^iv^	0.34 (12)	O1—S1—O3—K1^ii^	148.8 (4)
O1W^ii^—K1—O2—S1	52.9 (3)	O2—S1—O3—K1^xi^	−124.6 (2)
O1W^ii^—K1—O2—K1^iv^	−77.01 (13)	O2—S1—O3—K1^ii^	19.2 (5)
O3^ii^—K1—O2—S1	4.4 (4)	C1—S1—O3—K1^xi^	121.20 (17)
O3^ii^—K1—O2—K1^iv^	−125.51 (18)	C1—S1—O3—K1^ii^	−95.0 (5)
O2^iii^—K1—O2—S1	−117.8 (3)	K1^xi^—S1—O3—K1^ii^	143.8 (5)
O2^iii^—K1—O2—K1^iv^	112.39 (14)	O1—S1—C1—C2	−59.9 (4)
O2—K1—S1^i^—O1^i^	170.27 (18)	O1—S1—C1—C6	121.7 (4)
O2—K1—S1^i^—O2^i^	74.8 (2)	O2—S1—C1—C2	61.1 (4)
O2—K1—S1^i^—O3^i^	−15.2 (2)	O2—S1—C1—C6	−117.4 (4)
O2—K1—S1^i^—C1^i^	−99.86 (19)	O3—S1—C1—C2	−179.4 (4)
O1W—K1—O1^i^—S1^i^	175.60 (18)	O3—S1—C1—C6	2.2 (4)
O2—K1—O1^i^—S1^i^	−12.0 (2)	K1^xi^—S1—C1—C2	−123.1 (3)
O2—K1—O3^i^—S1^i^	165.0 (2)	K1^xi^—S1—C1—C6	58.5 (4)
O2—K1—O3^i^—K1^iv^	−0.34 (12)	S1—C1—C2—I1	2.7 (6)
O1W—K1—O1W^ii^—K1^ii^	0.00 (11)	S1—C1—C2—C3	−178.3 (4)
O2—K1—O1W^ii^—K1^ii^	−85.89 (11)	C6—C1—C2—I1	−178.9 (4)
O1W—K1—O3^ii^—S1^ii^	63.9 (4)	C6—C1—C2—C3	0.1 (7)
O1W—K1—O3^ii^—K1^iii^	−80.44 (12)	S1—C1—C6—C5	178.1 (4)
O2—K1—O3^ii^—S1^ii^	13.9 (6)	C2—C1—C6—C5	−0.4 (8)
O2—K1—O3^ii^—K1^iii^	−130.4 (2)	I1—C2—C3—C4	179.6 (4)
O1W—K1—O2^iii^—K1^iii^	73.96 (12)	C1—C2—C3—C4	0.5 (7)
O1W—K1—O2^iii^—S1^iii^	−152.5 (3)	C2—C3—C4—C5	−0.8 (9)
O2—K1—O2^iii^—K1^iii^	153.89 (12)	C3—C4—C5—C6	0.6 (9)
O2—K1—O2^iii^—S1^iii^	−72.6 (3)	C4—C5—C6—C1	0.1 (9)

Symmetry codes: (i) −*x*, *y*−1/2, −*z*+3/2; (ii) −*x*, −*y*+1, −*z*+2; (iii) *x*, −*y*+1/2, *z*+1/2; (iv) *x*, −*y*+1/2, *z*−1/2; (v) −*x*+1, *y*−1/2, −*z*+3/2; (vi) *x*, *y*, *z*−1; (vii) *x*, *y*, *z*+1; (viii) *x*, −*y*+3/2, *z*−1/2; (ix) −*x*+1, *y*+1/2, −*z*+3/2; (x) *x*, −*y*+3/2, *z*+1/2; (xi) −*x*, *y*+1/2, −*z*+3/2.

### Hydrogen-bond geometry (Å, °)

**Table:**

*D*—H···*A*	*D*—H	H···*A*	*D*···*A*	*D*—H···*A*
O1W—H1WB···O1^vii^	0.86	2.03	2.855 (5)	160
O1W—H1WA···O3^ii^	0.86	2.41	3.266 (5)	179
C6—H6···O3	0.93	2.42	2.834 (6)	107

Symmetry codes: (vii) *x*, *y*, *z*+1; (ii) −*x*, −*y*+1, −*z*+2.
